# Bacterial-Derived Polyhydroxyalkanoate/Bioceramic Composites in Clinical Practice: State of the Art and Future Perspectives

**DOI:** 10.1021/acsbiomaterials.5c00407

**Published:** 2025-07-21

**Authors:** Ewelina Cichoń, Maciej Guzik

**Affiliations:** Jerzy Haber Institute of Catalysis and Surface Chemistry, Polish Academy of Science, Niezapominajek 8, Krakow 30-239, Poland

**Keywords:** bioceramics, polyhydroxyalkanoates, composites, sustainability

## Abstract

This review examines the current advancements in and potential of bioceramic/polyhydroxyalkanoate (BioC/PHA) composites, emphasizing their growing role in biomedical applications. The integration of PHAs—biodegradable, biocompatible polymers from a bacterial origin—with bioceramics like hydroxyapatite or bioglass offers a unique synergy, combining the structural integrity of ceramics with the tunable properties of PHAs. Such composites demonstrate significant promise in bone tissue engineering, cartilage repair, and drug delivery systems, where they support cell attachment, proliferation, and targeted therapeutic release. The review highlights various methods of manufacturing these composites. Additionally, the review addresses challenges in production scalability, cost, and material purification necessary to meet medical-grade standards. Advances in functionalization, such as drug incorporation and bioactive coatings, are discussed as pathways to customized therapeutic solutions. This review underscores the transformative potential of BioC/PHA composites in creating sustainable, multifunctional biomaterials that align with the clinical demands of regenerative medicine and environmentally conscious material science.

## Graphical abstract

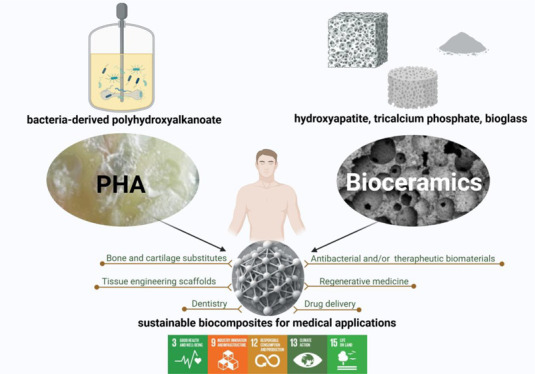

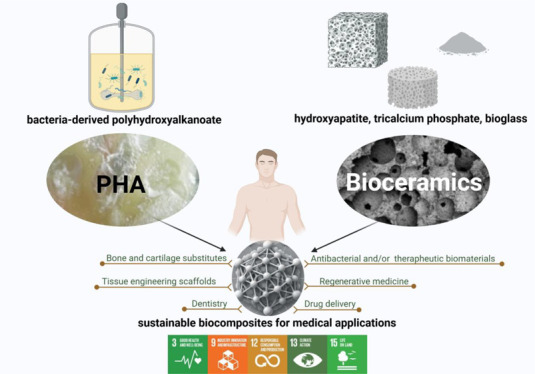

## 1 Introduction

Biomaterials interact with biological systems and are designed for use in contact with cells, tissues, and body fluids. According to the European Society for Biomaterials, a biomaterial is defined as a “material intended to interface with biological systems to evaluate, treat, augment, or replace any tissue, organ, or function of the body”. Biomaterials play an increasingly crucial role in tissue replacement. Synthetic substitutes offer alternatives to the limited supply of autografts and help address the issues associated with allogenic and xenogenic grafts.^1^ The rapid advancements in biomedicine, tissue engineering, gene therapies, and controlled-release drug delivery have highlighted the limitations of traditional single-component materials (e.g., metals, ceramics, and polymers) in meeting the complex demands of modern biomaterials.^2^ Consequently, composite materials are gaining popularity, as they provide enhanced functionality and versatility.

Composites are engineered to achieve materials with synergistic or enhanced properties beyond those of individual components. In bioceramic–polymer composites, the matrix role—whether polymer or ceramic—varies depending on the application. For medical applications, bioceramics are often preferred as they include ceramics compatible with the human body. Bioceramics are typically classified as nearly inert (e.g., alumina and zirconia), bioactive (e.g., hydroxyapatite and bioglasses), or bioresorbable (e.g., tricalcium phosphate). The latter two types are used to encourage apatite formation, a desirable property for bone substitutes due to their chemical similarity to the mineral phase of natural bone.

However, ceramic materials present certain limitations including brittleness, low tolerance to dynamic loads, low fracture energy, and slow degradation. These physical drawbacks restrict ceramics’ applications, but they can be mitigated by forming composites with polymers. Adding polymers to a ceramic matrix helps tailor the material’s properties to meet the requirements of regenerating tissue, such as providing adequate elasticity at the cell/polymer/ceramic interface. Conversely, ceramic particles are often used as fillers in polymer matrices to enhance the bioactivity or reinforce the mechanical strength. By selection of an appropriate polymeric component and adjustment of the organic/inorganic ratio, biomaterials can be customized for a range of applications. Bioceramic/polymer composites are intended to replicate the composite structure of natural bone or other targeted tissues.

The primary aim of this review is to consolidate recent advancements and provide insights into bioceramic/polyhydroxyalkanoate (BioC/PHA) composites with potential applications in medicine. The article describes the role of bioceramics as components in polymer/ceramic composites, highlighting their contribution to composite performance. The advantages of PHAs and their current medical applications are examined. The main section presents a comprehensive overview of the existing literature on BioC/PHA composites followed by a discussion of future research directions in this field.

## 2 Polymer/Bioceramic Composites Applied in Medicine

Bioceramic/polymer composites have garnered significant attention due to their biocompatibility, nontoxicity, and potential for delivering drugs and biomolecules.^3,4^ Numerous ceramic/polymer composites have been developed for medical applications.^5–8^ Manufacturing methods for these composites are also continually advancing.^9–13^ The primary application of bioceramics in the medical field is in bone tissue engineering^14–17^ where calcium phosphates (CaPs) and bioglasses are the most widely studied due to their strong bioactivity potential. Their usefulness in medical applications has led to the publication of over 11,000 studies on bioceramics (Figure 1B).

**Figure 1:**
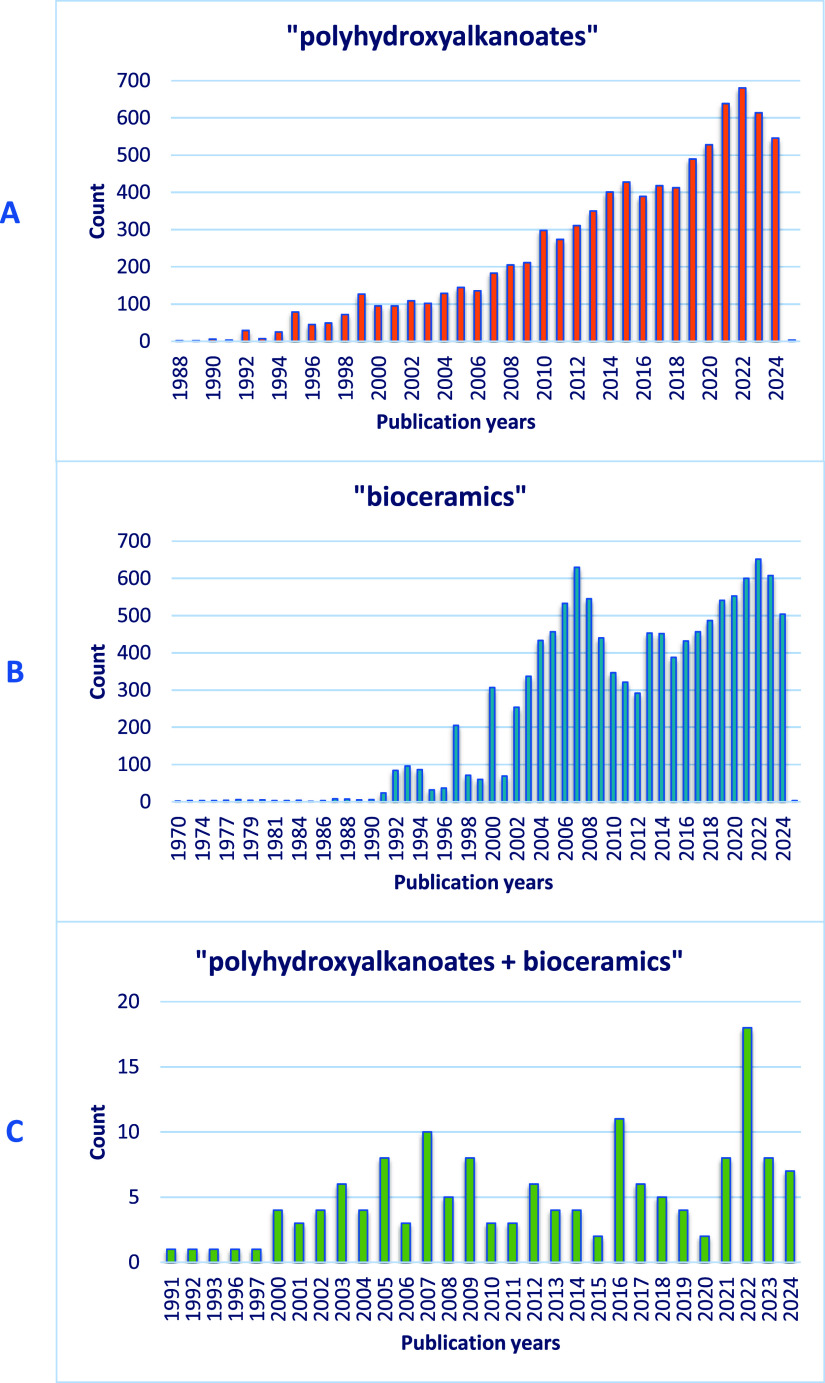
Number of papers in individual years in the subject of (a) “bioceramics”, (b) “polyhydroxyalkanoates”, and (c) “polyhydroxyalkanoates + bioceramics” according to Web of Science (12.11.2024).

Biodegradable polymers used in medical applications include synthetic polyesters, such as poly­(lactic acid) (PLA), poly­(glycolic acid) (PGA), and poly-ε-caprolactone (PCL), as well as natural polymers, such as collagen, hyaluronic acid, albumin, agarose, alginate, chitosan, cellulose, starch, dextran, polyamino acids, and polyhydroxyalkanoates—the primary focus of this review. Generally, porous implants are preferred over dense ones for bone substitutes, as interconnected porosity allows tissue to grow into the implant and promotes conditions favorable for neovascularization.^18^ Beyond traditional applications, bioceramics are also used in the engineering of smart materials for biomedical purposes. For instance, incorporating various calcium phosphates or bioglasses into polymer matrices can buffer the acidic byproducts formed during the hydrolytic degradation of resorbable polymers. In smart materials, growth factors or extracellular matrix-like molecules are often added to further enhance functionality.^19,20^

### 2.1 Manufacturing Techniques of Bioceramic/Polymer Composites

A wide range of techniques have been developed for the fabrication of bioceramic/polymer composites, each offering specific advantages in terms of mechanical performance, structural integrity, porosity, and biological response.

#### 2.1.1 Simple Manufacturing Methods

Coating, blending, or infiltrating ceramic materials with polymers can enhance the properties of the base ceramic, particularly by improving mechanical performance.^21–24^ This enhancement is likely due to the polymer filling ceramic microcracks, which increases flexibility, compressive strength, and overall toughness compared to nonimpregnated ceramic scaffolds.^25–28^ One effective strategy to alter mechanical properties while preserving the interconnected porosity of ceramic scaffolds is polymer infiltration into an inorganic matrix. For example, Martínez-Vázquez et al.^29^ infiltrated β-tricalcium phosphate (β-TCP) scaffolds fabricated by robocasting with PLA and PCL. This infiltration increased uniaxial compressive strength by 3-fold with PCL and 6-fold with PLA while also enhancing structural integrity after initial cracking.

Bioceramic/polymer composites can also be produced by simply mixing ceramic granules with a polymer suspension. Borkowski et al.^30^ demonstrated this by coating hydroxyapatite (HAp) granules with the polymer β-1,3-glucan. The resulting composite samples exhibited high flexibility and could be compressed or bent to adapt to specific shapes. *In vivo* studies on alveolar bone augmentation showed that using a small amount of these granules effectively filled the cavity, with nearly complete healing at the implant site after approximately 2 weeks.

Foam generation is another promising method for manufacturing polymer/bioceramic scaffolds. Pek et al.^31^ created porous collagen–apatite nanocomposite foams by centrifuging the slurry prior to freeze-drying and cross-linking. These scaffolds closely resembled natural bone and demonstrated effective healing of critical-sized femoral gaps in Wistar rats and segmental bone defects in the tibias of Yorkshire–Landrace pigs.

Modern scaffolds formed from tangled fibers *via* electrospinning may offer additional benefits,^32^ especially when fibers are doped with mineralizing particles. Many researchers have explored this approach.^33,34^ For instance, Qi et al.^35^ developed poly­(l-lactic acid)/PCL/HAp nanofibrous scaffolds for bone tissue engineering, which showed increased osteoblast proliferation, alkaline phosphatase (ALP) activity, and osteocalcin concentration compared to scaffolds without HAp. In another study, Maheshwari et al.^36^ reinforced electrospun composite mats of poly­(vinyl alcohol) (PVA) and PCL with β-TCP. The inclusion of β-TCP not only enhanced the mechanical properties but also significantly improved the thermal stability of the nanocomposites.

Polymer/bioceramic materials have also been effectively applied in the manufacturing of bone cements. Unlike physical blends, these chemically bonded materials are created by mixing the powder and liquid phases. In polymer/calcium phosphate bone cements, the ceramic is typically part of the powder phase, while polymers can be added to either phase. Ambrosio et al.^37^ introduced a PVA water solution to calcium phosphate powder (98% α-TCP, 2% HA) to create an injectable bone cement. Adding PVA increased the setting time due to the liquid phase’s higher viscosity. When implanted in the cancellous bone of New Zealand white rabbits, the composite showed a faster healing response, as evidenced by a greater trabecular tissue penetration. At the bone–composite interface, newly formed bone exhibited superior mechanical properties compared with the bone–cement interface. Pańtak et al.^38^ developed innovative hybrid biomicroconcrete-type composites using α-tricalcium phosphate, HAp-chitosan granules, and natural polysaccharides, with a citrus pectin gel as the liquid phase. The developed cementitious materials demonstrated high plasticity, cohesion, and bioactivity, making them promising candidates for filling complex-shaped bone tissue defects.

One primary limitation of calcium phosphate bone cements is their low porosity. Roy et al.^39^ addressed this by developing a bone cement with tunable macro- and microporosity for controlled drug delivery. Mannitol was used as a porogen to create micropores, while poly­(lactic-*co*-glicolic) acid (PLGA) microspheres were incorporated for macroporosity. They studied *in vitro* vancomycin release from cements containing vancomycin in three configurations: embedded in CaP powder, encapsulated in PLGA microspheres, and within a PLGA-CaP mixed powder. Release studies showed rapid antibiotic release from CaP powder alone, while the most controlled release was achieved in calcium phosphate bone cement containing vancomycin-loaded PLGA microspheres.

#### 2.1.2 Composites Obtained Using Additive Manufacturing Techniques

Additive manufacturing techniques are frequently employed to achieve the desired porosity in materials, an essential property that facilitates body fluid transport through porous implants.^40^ The recent literature suggests that these techniques offer a fast and economical alternative for producing “on-demand” scaffolds for biomedical applications.^41,42^

Russias et al.^43^ utilized hybrid inks for scaffold printing *via* the robocasting technique using PLA or PCL dissolved in methylene chloride mixed with HAp, bioglass 45S5, or bioglass 6P53B powders. However, they found that the hygroscopic nature of the 45S5 bioglass prevented the development of an ink optimized for rapid prototyping. In another study, Corcione et al.^44^ prepared an HAp/PLA filament for 3D printing of a porous maxillary sinus model using fused deposition modeling. HAp powder was used as a ceramic filler within the PLA matrix—a popular biodegradable polymer in biomaterials. In their method, PLA pellets were uniformly coated with an HAp layer, melted using a rotomolding machine to avoid toxic solvents, and formed into a filament. This procedure resulted in good dispersion of HAp particles within the polymer matrix, with the composite showing a slight increase in flexural modulus compared to pure PLA.

Suwanprateeb with collaborators^45^ enhanced the mechanical properties of a 3D-printed HAp scaffold by infiltrating it with PCL of low and high molecular weights. Infiltration with a single type of PCL (either low or high molecular weight) improved the flexural modulus compared to noninfiltrated HAp. However, sequential infiltration with both low- and high-molecular-weight PCL led to even greater increases in the flexural modulus, strength, and energy at break. This combined infiltration approach also positively affected the biological properties of the scaffolds, supporting high osteoblast proliferation and differentiation at levels comparable to those observed with pure HAp.

#### 2.1.3 Functional Composites: Drug Delivery Systems and Membranes

In addition to enhancing toughness in inorganic/polymer composites, incorporating a soft biodegradable polymer into a bioactive ceramic scaffold enables the controlled delivery of therapeutic drugs or other bioactive molecules. Polymer/inorganic composites are, therefore, highly applicable in drug delivery systems. Wu et al.^46^ proposed polymer/inorganic hybrid nanoparticles to effectively counteract cancer drug resistance. In their study, biotin-heparin/heparin/calcium carbonate/calcium phosphate hybrid nanoparticles were used as drug carriers. The biotin-heparin component facilitated biotin-mediated tumor targeting, while the inorganic compounds improved drug loading and release due to their nanoporous structure. Another example of a bioceramic/polymer drug carrier was described by Martins et al.,^47^ who encapsulated the anticancer drug paclitaxel in a bionanocomposite formed by Mn–Zn ferrite nanoparticles with magnetic properties, designed for targeted cancer therapy. To enhance biocompatibility, these nanoparticles were coated with chitosan, and the surface was further modified with nano-HAp. In addition, hormones, such as oxytocin, can be delivered by polymer/bioceramic composites.^48^ A hydrogel polymer scaffold embedded with spherical oxytocin and biphasic calcium phosphates improves calvarial bone regeneration in rats.^49^

Dumont et al.^50^ prepared composite membranes using a one-step coprecipitation method in water, embedding nano-HAp particles within chitosan and *O*-carboxymethyl chitosan matrices. These hybrid systems were found to be noncytotoxic to a human osteoblast-like cell line and show promise as biomaterials for potential cartilage and bone tissue repair and replacement. Dziadek et al.^51^ investigated tunable PCL-based membranes modified with bioactive glass (BG) particles, derived from gel, in two sizes (<45 and <3 μm). The composite membranes exhibited excellent bioactivity, confirmed by incubation in simulated body fluid, with the results showing that the bioactivity kinetics could be controlled by the preparation method and BG particle size.

#### 2.1.4 Discussion on Manufacturing Methods of Bioceramic/Polymer Composites

Collectively, the manufacturing technique for bioceramic/polymer composites plays a determining role in the properties of the final material, such as mechanical strength, porosity, degradability, and bioactivity. Infiltration of polymers into prefabricated ceramic scaffolds is one of the straightforward methods with an improved mechanical enhancement in brittle ceramics while maintaining their porosity. This method is specific for load-bearing applications and can be customized by choosing polymers of varying stiffnesses or degradation rates. However, the reproducibility of infiltration and full pore penetration is still a concern, particularly in scaffolds with a complicated architecture.

Physical mixing and the polymeric coating of ceramic particles are economically viable techniques. Products from these processes are marked by excellent flexibility and biocompatibility, but they are limited by poor mechanical strength and inadequate structural integrity upon dynamic loading.

Electrospinning yields highly porous, fibrous scaffolds with good surface-to-volume ratios conducive to cellular attachment, growth, and differentiation. Electrospun membranes can also be compounded with nano- or microparticles of bioceramics for the development of osteoconductive properties. However, electrospinning is limited when large 3D structures are needed and typically requires postprocessing or layering techniques in the production of scaffolds for clinical relevance.

Foam generation techniques, particularly freeze-drying and cross-linking of polymer–ceramic slurry, are commonly employed to replicate the trabecular bone structure. Materials in the form of foams enhance vascularization and cell ingrowth as a result of their open structures and large pore sizes. Despite their biological advantages, their mechanical instability limits their application to non-load-bearing areas or as bioactive fillers but not as structural implants.

Bone cements, developed through the chemical combination of ceramic powders and liquid phases containing polymers, possess unique benefits of injectability, moldability, and an often quick *in situ* setting. They can be used in minimally invasive surgery and can be utilized for prosthesis anchorage or filling tiny bone voids. Incorporation of porogens or biodegradable polymer microspheres provides controlled drug delivery or increased porosity to widen their clinical uses. Nevertheless, conventional cements are compromised by their poor mechanical performance and long-term integration.

Additive manufacturing techniques offer high reproducibility and precision, enabling the consistent fabrication of scaffolds with controlled architecture and porosity across multiple batches. However, the design and optimization of such scaffolds often require considerable time investment, including detailed CAD modeling and parameter tuning. Moreover, achieving high resolution and dimensional accuracy typically necessitates the use of advanced, high-end equipment, which can be cost-prohibitive.

Following this, hybrid approaches comprising additive manufacturing with subsequent polymer infiltration or surface treatment are very promising. They allow for the independent manipulation of mechanical and biological properties, with the potential to create graded or multiphasic scaffolds that reflect the hierarchical structure of natural bone. In summary, while no single technique is universally superior to the rest, the choice of a manufacturing method should be guided by the particular application—whether the primary intent is structural reinforcement, defect repair, drug delivery, or biological integration. The growing trend toward the combination of traditional methods with computer-assisted fabrication and smart material design is leading to the development of next-generation bioceramic/polymer composites tailored to treat specific clinical problems.

As demonstrated, bioceramic/polymer composites are promising candidates for medical applications, with many already in use. These composites are primarily applied in bone tissue engineering, dentistry, and drug delivery systems. Additional examples of polymer/bioceramic composites are provided in Table 1.

**Table 1:** Polymer/Bioceramic Composites: Synthesis Methods and Applications in Biomedical Engineering

**used polymer**	**inorganic component**	**obtaining method**	**composite form**	**application**	**limitations**	**research model**	**reference**
acrylamide, ammonium polyacrylate	α-TCP	3D printing	3D-printed cementitious scaffolds (bars and cylinders)	scaffolds for cellular growth and cancellous bone replacement	not suitable for load-bearing applications	no biological/cellular model used	^52^
collagen	β-TCP	β-TCP dispersion injected into a Terudermis scaffold	commercially available scaffold modified with ceramic nanoparticles	3D scaffolds for bone tissue engineering	high doses of nanoparticles caused inflammation, reduced bone formation, and limited cell/tissue infiltration	*in vitro* (MC3T3-E1 cells), *in vivo* (rat subcutaneous tissue and cranial bone defect model)	^53^
gelatin, PVA, PCL	HAp	3D printing and polymer infiltration	3D-printed scaffolds infiltrated with polymer	scaffolds for regenerative medicine	infiltration mostly limited to outer layers; insufficient mechanical stability in body-like environments	no biological/cellular model used	^54^
maltodextrin	tetracalcium phosphate, dicalcium phosphate anhydrous	cement mixing and setting	cement with tubular macropores generated by maltodextrin microstrips	macroporous bone cements, bone tissue engineering	high porogen content (30%) delays setting time and reduces HAp conversion and mechanical strength; not suitable for load-bearing applications	*in vitro* (MC3T3-E1 cells)	^55^
PCL, PLA	β-TCP	robocasting, polymer infiltration	robocast scaffolds infiltrated with polymer	3D scaffolds for bone tissue engineering	not suitable for load-bearing prior to infiltration; PLA-infiltrated scaffolds reached cortical bone strength	no biological/cellular model used	^29^
PLA	bioglass	polymer mixed with BGparticles	disc-shaped polymer-based composites with bioglass; diameter 5 mm, thickness 1 mm	disc-shaped polymer/bioglass scaffolds supporting the function and survival of endothelial progenitor cells *in vitro*; they can be used in bone tissue engineering	designed for early vascularization; not suitable for load-bearing; tested only short-term (1 week)	*in vitro* (human endothelial progenitor cells), *in vivo* (rat critical-size calvarial defect)	^56^
silanized hydroxypropyl methylcellulose	α-TCP	cement mixing (ceramic powder and polymer solution) and setting	injectable paste which sets	calcium phosphate bone cement with better injectability as well as rheological and mechanical properties	not suitable for load-bearing due to limited strength at low liquid to powder ratios; air entrapment may reduce strength	no biological/cellular model used	^57^
gellan gum	α-TCP	foam replica method, sintering and then immersion in polymer solution	porous ceramic scaffold coated with polymer	bioceramic scaffold with enhanced compressive strength for bone tissue engineering	not suitable for load-bearing	no biological/cellular model used	^58^
PLGA	silver containing TCP	electrospinning of polymer solution with dispersed ceramic nanoparticles	electrospun nanofabric scaffold	nanofabric scaffold; antibacterial regenerative pulp-capping material	cytotoxic to human dental pulp cells; reduced viability and proliferation; strong proinflammatory response	*in vitro* (human dental pulp cells)	^59^
chitosan	β-TCP	freeze-drying	polymer foam decorated with ceramic particles	composite scaffold supporting higher proliferation rate of human periodontal ligament cells and recruiting vascular tissue ingrowth in comparison with chitosan scaffold	not suitable for load-bearing	*in vitro* (human periodontal ligament cells), *in vivo* (subcutaneous implantation in athymic mice)	^60^
cellulose acetate, gelatin	HAp	electrospinning of polymer solution with dispersed ceramic nanoparticles	electrospun nanofibrous mat	nanocomposite mats for wound dressing applications	reduction in tensile strength with higher HAp content; polymer + 50 mg HAp showed signs of foreign body reaction	*in vitro* (L929 fibroblasts); *in vivo* (rat full-thickness skin wound model)	^61^
PCL	α-TCP	electrospinning of polymer solution with dispersed ceramic particles	electrospun fibers with ceramic nanopowder (nanofibrous membranes)	electrospun membranes for clinically relevant constructs for bone tissue engineering.	high α-TCP content (2 wt %) reduces mechanical strength due to nanoparticle agglomeration	no biological/cellular model used	^62^

### 2.2 Various Formulations of Bioceramic/Polymer Composites and Their Impact on Physicochemical and Biological Properties

The formulation of bioceramic/polymer composites plays a crucial role in determining their physicochemical and biological performance in biomedical applications. These materials aim to integrate the bioactivity of ceramics with the flexibility, degradability, and processability of polymers, yet the balance between these phases must be carefully tuned to avoid detrimental effects.

When the polymer serves as the matrix, excessive ceramic content often leads to particle agglomeration, resulting in an inhomogeneous microstructure and compromised mechanical integrity. This phenomenon is particularly problematic for nanoparticles, which tend to cluster in hydrophilic polymers unless surface modification or dispersants are used.^63^ Although ceramic fillers increase stiffness (Young’s modulus) in dry conditions, their reinforcing effect may diminish in aqueous environments due to hydrophilicity or poor interfacial bonding. Therefore, optimal reinforcement requires homogeneous dispersion and control over the ceramic particle surface properties. At the same time, the ceramic phase should be present in sufficient quantity to ensure functional bioactivity, such as promoting mineralization, osteogenesis, or an antibacterial response. Inadequate ceramic loading diminishes such effects.

Conversely, in systems where ceramics act as the structural framework (e.g., ceramic scaffolds coated with polymer), polymer incorporation must be carefully adjusted. Elastic polymers surrounding the ceramic strut improve toughness and resistance to fragmentation, especially in applications involving dynamic stress.^25^ Regarding coating thickness, a thicker polymer layer can hinder ion exchange and sometimes reduce bioactivity, such as in the case of silicate ion release from composite films containing high concentrations of polyphenolic compounds.^64^ It may also enhance biocompatibility by providing a more favorable surface for cell adhesion and proliferation, as observed for PLGA-coated silicate scaffolds.^65^ Surface roughness and mechanical cues should also be taken into account when designing bioceramic/polymer composites as they influence cellular response.^66^ For instance, bone or epithelial cells typically require substrates of specific stiffness and porosity to promote growth and differentiation.^67^ Thus, the optimal thickness of the polymer layer should be carefully balanced based on the intended biomedical application.

Importantly, the choice of polymer must account for the degradation behavior. Natural polymers (e.g., chitosan and carboxymethyl cellulose) often degrade more rapidly than synthetic ones (e.g., PLA and PCL), which can be advantageous for drug delivery but problematic for long-term implants.^68^ Moreover, when biologically active agents (e.g., polyphenols, growth factors, and antimicrobials) are embedded, their release kinetics is heavily influenced by both polymer degradation and the composite microstructure.^69^

In conclusion, the physicochemical and biological properties of bioceramic/polymer composites are highly dependent on formulation parameters including phase ratios, interface engineering, and degradation behavior. Fine-tuning these variables enables the design of biomaterials tailored for specific biomedical applications.

## 3 Polyhydroxyalkanoates: Polymers of the Future

Bacteria-derived polymers such as dextran, gellan gum, or polyhydroxyalkanoates are interesting alternatives to oil-based or petroleum ones, as they are nontoxic and biocompatible. Polyhydroxyalkanoates are bacterial carbon and energy reserve materials of widespread occurrence. The presence of PHA in microbial cells is the response to environmentally challenging conditions.^70^ These polyesters can be synthesized by at least 100 different bacterial species in response to an environmental stress. From a material scientist’s point of view, PHAs are biodegradable polyesters with properties that can be tailored, including stiffness, crystallinity, elasticity, and degradation rate. These characteristics are influenced by the polymer’s monomeric composition, microbial strain, substrate selection, production process parameters, and postprocessing treatments, including extraction methods. The ability to obtain polymers with specific properties has driven researchers to employ genetic engineering and innovative fermentation techniques, expanding the range of available PHA types. These biopolymers are an important alternative to petroleum-based polymers due to their environmentally friendly production processes and biodegradability.^71,72^ Interest in PHAs has surged due to their biodegradability, biocompatibility, and versatility, which make them valuable across multiple fields. Although initially focused on packaging, PHA applications have expanded to include medicine, agriculture, and wastewater treatment, driven by their adaptable thermoplastic properties that allow production in forms such as foams, fibers, coatings, and microspheres.^73^ This versatility has fueled over 6500 publications on PHAs (Figure 1A), with current research employing advanced genetic engineering and fermentation techniques to broaden the array of available PHAs. The first commercial PHA product, introduced by Wella AG in 1990, marked a milestone in the commercialization of bioplastics and set the stage for further development across diverse sectors.^74^

### 3.1 Historical Background and Classification

Poly­(3-hydroxybutyrate) (PHB) is the simplest of PHAs and belongs to scl-PHAs. Polyhydroxyalkanoates comprise a diverse group of biopolymers produced by bacteria, primarily composed of 3-hydroxyacids formed through bacterial metabolic processes. These materials can be classified into two main groups based on the length of carbon atoms in each monomer unit. The first group, short-chain-length PHAs (scl-PHAs), includes PHAs with side chains of three to five carbon atoms, such as PHB and poly­(3-hydroxyvalerate) (PHV). scl-PHAs are characterized by their rigidity and brittleness, with high crystallinity and melting points ranging from 160 to 180 °C.^75^ PHB, in particular, is noted for being the most rigid PHA, but it has limited processability and a rapid aging rate, factors that may limit its range of applications. The second group, medium-chain-length PHAs (mcl-PHAs), includes PHAs with side chains containing 6 to 14 carbon atoms, imparting greater flexibility and lower crystallinity compared to scl-PHAs. This group also has lower melting points, and its members, such as poly­(3-hydroxyhexanoate) (PHHx), poly­(3-hydroxyoctanoate) (PHO), and poly­(3-hydroxynonanoate) (PHN), are suitable for applications that require more pliable materials. PHAs can also be synthesized as either homopolymers, containing only one type of monomer, or copolymers, incorporating two or more types of monomers within the polymer chain. Copolymers such as poly­(3-hydroxybutyrate-*co*-3-hydroxyvalerate) (PHBV) and poly­(3-hydroxybutyrate-*co*-3-hydrohexanoate) (PHBHHx) provide tailored properties, enhancing their versatility for various applications.

### 3.2 Derivation of Polyhydroxyalkanoates from Bacterial Species

Maurice Lemoigne in 1926 first reported the accumulation of PHB in .^76^ Since then, numerous bacterial strains capable of PHA production have been identified across diverse ecological niches—from wastewater and marine environments to hydrocarbon-contaminated soils. Microorganisms capable of producing PHA can store these polymers at levels reaching up to 90% of their cell dry weight.^77^ Only a limited number of bacterial strains within each species have been extensively characterized and successfully scaled up for industrial production. Among the bacterial strains studied for industrial-scale PHA production, KT2440 is one of the most prominent. This Gram-negative strain is valued for its high metabolic versatility, genetic tractability, and ability to accumulate up to 75% mcl-PHA per cell dry weight, especially when grown on fatty acids.^78^ Another efficient mcl-PHA producer, ATCC 29347, utilizes *n*-alkanes and long-chain fatty acids, generating C6–C14 monomers *via* β-oxidation.^79^ shows excellent potential for converting lignocellulosic biomass into PHB, achieving yields up to 80% of its cell dry weight from sucrose.^80^ Finally, H16 is a model PHB producer capable of both heterotrophic and chemolithoautotrophic growth. It can utilize a wide range of substrates and reach high cell densities, making it particularly attractive for sustainable bioplastic production. Under optimized fed-batch conditions, it can reach biomass concentrations up to 164 g/L with PHB content exceeding 76% of cell dry weight and productivity around 2–3.1 g·L^–1^·h^–1^, depending on the setup.^81^ Even halophilic organisms such ascan synthesize PHA in high-salt media using unrefined feedstocks like glycerol or molasses, eliminating the need for sterile conditions and reducing production costs.^82^ The yield of PHA production depends on the strain, cultivation conditions, and the carbon to nutrient ratio. Although pure cultures offer high yields and product uniformity, mixed microbial cultures (MMCs) have gained attention as a cost-effective alternative that can utilize industrial or municipal waste streams.^83^ However, MMCs typically achieve a lower PHA content in biomass due to microbial diversity and competition.

PHA extraction from microbial biomass is a critical downstream step that strongly influences both the purity of the final biopolymer and the overall economic feasibility of the production process. While mcl-PHAs can be easily extracted by “soft” solvents such as acetone or ethyl acetate, for scl-PHAs such as PHB, mainly halogenated solvents like chloroform have been used due to their high efficiency in dissolving or isolating PHA, achieving purities in the range of 86–99% and recovery rates up to 94%.^84^ However, their high toxicity and environmental impact limit their applicability. To address their drawbacks, various nonhalogenated alternatives have been tested for PHA extraction, including industrial solvents like anisole, phenetole, and cyclohexanone, which have shown promising results for PHB recovery.^85^ The digestion method is a widely studied approach for recovering PHA, aiming to selectively dissolve the non-PHA cell mass while leaving the polymer granules intact. Early techniques used strong oxidizing agents like sodium hypochlorite or sodium hydroxide, achieving recovery rates up to 90% but often causing partial degradation of PHA if concentrations were not carefully controlled.^86^ Mechanical methods like bead milling and high-pressure homogenization offer solvent-free disruption but are costly and less scalable.^87^ Advanced approaches like supercritical CO_2_ extraction, enzymatic digestion, solvent gelation, ultrasound-assisted extraction, and ionic-liquid-based methods provide further options, balancing yield, purity, and environmental impact, though often limited by cost or technical complexity.

### 3.3 Polyhydroxyalkanoates as Biomaterials

Recent studies have shown that certain types of PHAs exhibit improved biocompatibility, promoting bone cell growth and differentiation.^88^ They can be shaped in various forms such as foams, fibers, coatings, as well as nanoparticles. Because of their biocompatibility, they have also found wide use in medical applications such as sutures, dressings, orthopedic nails, marrow bone scaffolds, drug carriers, artificial valves, and scaffolds for bone and cartilage tissue (Table 2). They can be used also as a substrate for protein immobilization.^89^ In addition, their degradation products have been studied for pharmaceutical applications.^90^ A feature that differentiates them from other biodegradable polymers such as PLA or PGA is their degradation by surface erosion, not by bulk hydrolysis.^91^ This is a very important aspect for using them as drug carriers because of the wider control of drug release. PHAs are known for their hydrophobicity;^92^ however, wettability can be improved, for example, by carboxyl ion implantation.^93^ This method has the advantage of modifying only the polymer’s surface without altering its bulk properties. Another advantage is that PHAs’ properties can be tailored with a wide range of building blocks—monomers.^94^ For example, a PHA incorporating scl monomers like hydroxybutyrate (HB) and mcl monomers exhibits significantly improved mechanical properties compared to standard PHB.^95^

**Table 2:** Polyhydroxyalkanoates and Their Potential Applications in Medicine^97^

application site	PHAs used
bone tissue	PHBHHx, PHB, PHBV
cartilage tissue	PHBV, PHBHHx, PHB/PHO blend
blood vessels	PHB, PHBV, PHBHHx, PHO
heart valves, heart patches	PHBHHx, P4HB, PHO
peripheral nerves	PHB, PHBHHx, PHO/PHB blend, PHBVHHx
drug delivery systems	PHBHHx, PHB, PHBV, PHB/PHBHHx blend

Findings from numerous research groups suggest that PHA is a suitable material for medical devices, implants, and tissue engineering. To the best of our knowledge, no study has reported any carcinogenic effects induced by PHAs or their biodegradation products. According to the literature, PHAs tested so far are fully biocompatible, showing no toxicity to cells, tissues, or body fluids (such as blood),^96^ and can therefore be effectively used to produce biodegradable sutures, wound dressings, implants, cellular scaffolds, and drug delivery systems.^97^ Table 2 summarizes various polyhydroxyalkanoates and their potential medical applications. Unlike PGA or PLA, which can acidify their surroundings during degradation, polyhydroxyalkanoates maintain a stable pH, making them more compatible with cells and the immune system. TephaFLEX, an absorbable surgical suture made from poly-4-hydroxybutyrate (P­(4HB)), became the first U.S. Food and Drug Administration-approved polyhydroxyalkanoate product for medical use in 2007. Other commercially available PHA-based products include GalaFLEX and Phasix Mesh surgical meshes for reconstructive and plastic surgery, BioFiber for tendon reconstruction, and MonoMax Suture and Phantom Fiber surgical sutures.^98^

One of the primary advantages of PHAs over other biodegradable polymers is their ability to degrade under both aerobic and anaerobic conditions. PHAs can undergo thermodegradation or enzymatic hydrolysis. In biological systems, they are degraded by bacterial depolymerases in bacteria or in animal and human tissues through nonenzymatic or enzymatic hydrolysis.^99^ The rate of degradation depends on variables such as temperature, environmental pH, PHA shape and texture, molecular weight, crystallinity, and the presence of functional groups in the PHA structure.^100^ PHAs degrade mainly to (*R*)-3-hydroxy acids, which are natural metabolites found in the body, originating from the β-oxidation pathway.^101^ What distinguishes these polyesters from other biodegradable polymers is their degradation mechanism, which occurs by surface erosion rather than volumetric hydrolysis.^91^ This characteristic is highly desirable for drug delivery applications as it enables better control over the release of active substances. During PHA degradation, the polymer’s molecular weight decreases, while its crystallinity increases.^102^ As the degradation rate of PHAs is governed by a number of factors, their behavior can vary significantly depending on the application.^100^ Degraded PHAs may act as bacteriostatic agents, with both scl- and mcl-PHA proposed for bacterial infection control.^103^ PHA degradation products are utilized in the preparation of biodegradable, implantable rods that serve as antibiotic carriers for chronic osteomyelitis therapy.^104^ Additionally, PHB monomers are used in the synthesis of sex hormones,^105^ have a positive impact on osteoblast growth, exhibit antiosteoporotic effects, and enhance calcium deposition and serum ALP activity.^106^ Moreover, PHA-derived 3-hydroxy fatty acids are useful for producing medical devices such as suture fasteners, staples, screws, stents, and other applications.^107^ Furthermore, PHA degradation products nourish the surrounding tissues.^108^

Another advantage of PHAs is their ease of modification.^109^ This can be achieved among others by hydroxylation, carboxylation, or chlorination.^110^ Surface modifications of PHAs can include changes toward enhancing cell compatibility and antibacterial activity.^111^ In the study of Ladhari et al.,^112^ PHB microfiber membranes decorated with photoactive Ag-TiO_2_ nanoparticles demonstrate high antibacterial and antifouling efficacy, achieving over 99% bacterial elimination against and . The surface chemistry, wettability, and topography without alteration of the bulk properties can be adjusted to the proper application. Due to the presence of functional groups in their structure, PHAs and their oligomers offer numerous possibilities for attaching bioactive agents.^113^ Masood et al.^114^ grafted PHB and PHBV nanoparticles with folic acid. The obtained nanoparticles coated with poly­(ethylene glycol) and loaded with epirubicin demonstrated targeted and sustained drug delivery in MCF-7 cancer cells. Piddubnyak et al.^115^ presented the synthesis and toxicity evaluation of custom-designed oligo-[*R*,*S*]-3-hydroxybutyrates (OHBs) for potential drug delivery applications. These OHBs were tested on hamster V79 fibroblasts and murine melanoma B16­(F10) cells, showing no impact on cell viability after 96 h at 1–9 μg·mL^‑1^ and no cellular stress response in rat hepatoma cells. Furthermore, doxorubicin conjugated with OHBs was successfully internalized by melanoma cells, highlighting these biodegradable and biocompatible oligomers as promising, nontoxic drug delivery vectors. Haraźna et al.^116^ developed oligomeric conjugates of 3-hydroxy fatty acids and diclofenac for wound dressings, forming PHO patches using solvent casting/porogen leaching. These patches demonstrated antimicrobial activity against various bacterial strains and promoted HaCaT cell adhesion and proliferation, suggesting a strong potential for both normal and chronic wound treatment. Abdelmalek et al.^117^ chemically modified PHB, which was transformed to PHB-diethanolamine by transamination. Then, the chain ends of the polymer were substituted with caffeic acid molecules. The developed nanoparticles showed strong antibacterial activity, suggesting their potential for commercially active food coatings. Haraźna et al.^118^ explored the potential of PHO doped with tocopherol-modified layered double hydroxide nanoparticles as an active packaging material. The nanocomposite films demonstrated effective barrier properties for fruit and vegetable storage, inhibited growth, and helped preserve the polyphenol content in strawberries, suggesting promise as a sustainable alternative to extend shelf life and prevent bacterial contamination in food packaging.

Furthermore, PHAs in nanoparticulate form can be tailored for diverse biomedical uses, such as targeting cancer cells through the attachment of specific ligands, tracking them using fluorescent markers, or delivering drugs.^119^

The main challenge in applying PHAs as biomaterials is the cost of their production, which varies depending on the specific type of PHA; however, this factor is less critical in biomedical applications where high material purity and performance are prioritized. Although many polyhydroxyalkanoates have been utilized in biomedical applications, purification processes are essential to achieve medical-grade quality. Residual pyrogens, such as bacterial exotoxins and endotoxins left after polymer extraction, must be removed using mechanical or chemical methods (e.g., oxidation). Filtering polymer solutions in organic solvents through activated charcoal can effectively reduce endotoxin levels without damaging the PHA, although some polymer loss occurs.^120^ Alternatively, oxidizing agents like hydrogen peroxide or potassium permanganate can be used provided that they do not significantly degrade or alter the physical or chemical properties of the PHA.^121^ Additionally, biomaterials must be thoroughly sterilized to prevent infections; traditional methods such as ethylene oxide and gamma irradiation are effective for both sterilization and depyrogenation.^122^

## 4 Bioceramic/Polyhydroxyalakanoate Composites

Polyhydroxyalkanoates can be effectively utilized as components in composite materials.^123^ As polymers, PHAs are versatile and can be processed into various forms, including fibers, films, and foams, using techniques such as electrospinning and 3D printing. This adaptability enables the creation of scaffolds with specific architectures and properties tailored to diverse biomedical applications. Misra et al.^124^ reviewed research on polyhydroxyalkanoate/inorganic composites, highlighting the systems investigated, achieved microstructures, properties, and potential applications, with a special focus on tissue engineering scaffolds. The total number of papers in the Web of Science databases related to “polyhydroxyalkanoates + bioceramics” is only 151 (Figure 1C). In recent years, there has been a focus on integrating PHAs into ceramic–polymer biocomposites (Figure 2, Table 3), especially in developing novel scaffolds for bone tissue engineering.^125,126^ The inclusion of a low-modulus polymeric component acts as an “isoelastic medium”, effectively reducing the stiffness gradient between natural bone and a purely ceramic implant.^25^ PHAs’ high biocompatibility promotes cell attachment, proliferation, and differentiation, which are essential for implant integration with surrounding tissue and for supporting tissue regeneration.^127^ The use of these bacterial-origin polymers in bone tissue engineering offers new possibilities for the sustained nourishment of regenerating bone, as the (*R*)-3-hydroxy acids formed through PHA degradation stimulate osteoblast proliferation as nutrients for bone.^101^ Additionally, PHAs can be functionalized with bioactive compounds, such as drugs, offering promising applications due to their controllable degradation rate.^128^ This degradation rate can be adjusted by modifying the copolymer composition,^129^ allowing for better control over implant longevity and stability and ensuring that it degrades at a pace aligned with tissue regeneration needs.

**Figure 2:**
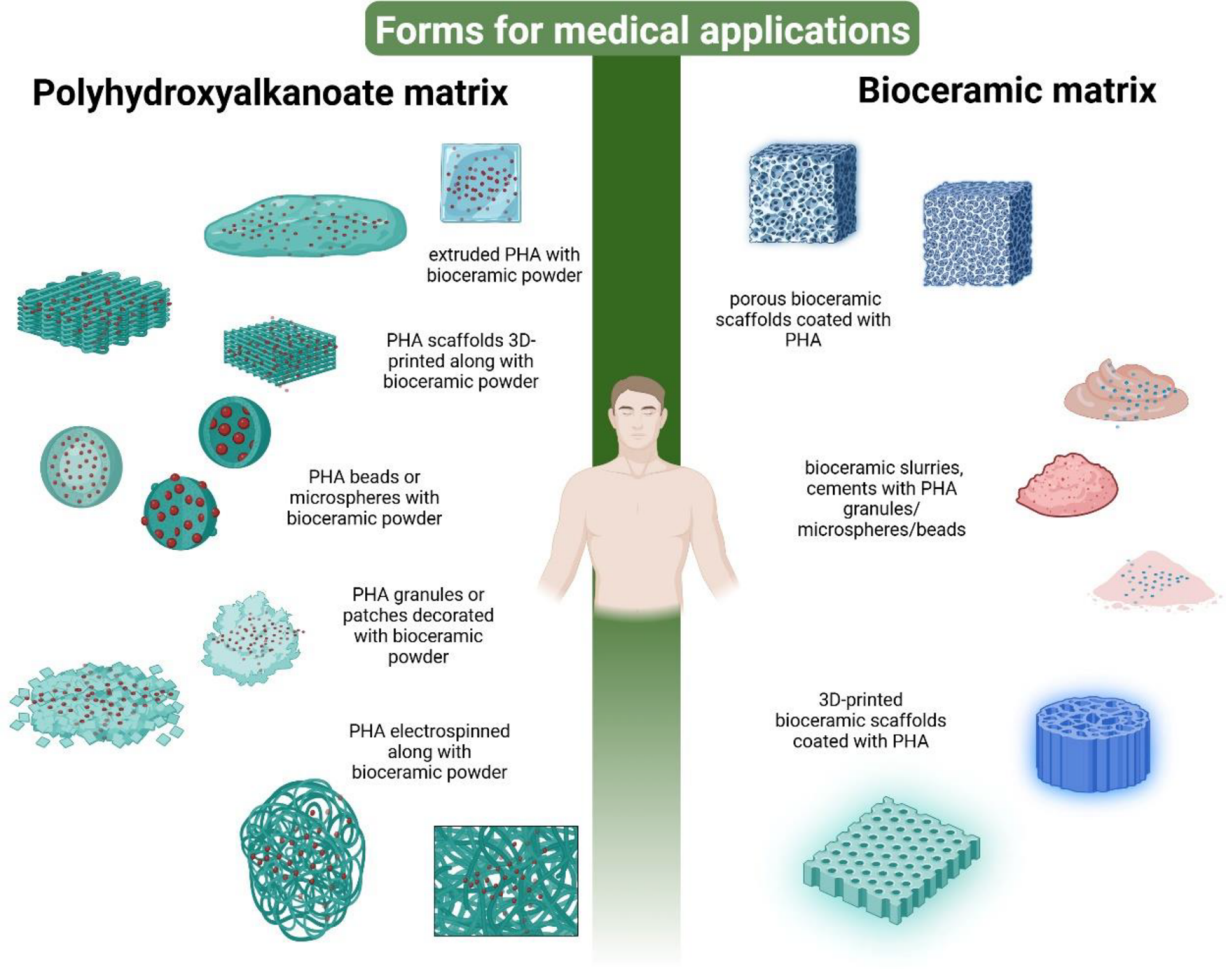
BioC/PHA composite forms for medical applications.

**Table 3:** BioC/PHA Composites and Their Applications in Biomedical Engineering.

**PHA used**	**ceramic component**	**method of fabrication**	**composite form**	**application**	**outcomes**	**research model**	reference
PHB	silver-decorated β-TCP	foam replica method, sintering, polymer infiltration	highly porous scaffolds	bone tissue engineering	coating improved compressive strength and surgical handling and promoted the slow release of (*R*)-3-hydroxybutyric acid and its oligomers, which may support tissue regeneration; suitable for non-load-bearing bone defects	no biological/cellular model used	^130^
PHB	porous β-TCP ceramics doped with silver/silicon	foam replica method, sintering, polymer infiltration	highly porous scaffolds	antibacterial scaffolds for bone regeneration	coating improved compressive strength; sustained antibiotic release for 120 h; strong antibacterial activity against , , and ; bioactivity confirmed by apatite formation; low *in vivo* toxicity, except for a slight reduction with the ceftazidime-loaded variant	*in vitro* (SBF immersion, antibacterial tests, drug release); *in vivo* (toxicity tested on )	^131^
PHB	nanobioactive glass	sol–gel synthesis of bioglass and electrospinning	electrospun nanofibrous scaffold	bone tissue engineering	7.5% bioglass addition improved tensile strength and promoted apatite layer formation after SBF immersion; higher bioglass contents reduced mechanical properties due to agglomeration; scaffold showed interconnected porosity	no biological/cellular model used; bioactivity assessment after immersion in SBF	^132^
PHB (with chitosan)	magnetic mesoporous bioglass	electrospinning polymer solution with dispersed ceramic nanoparticles (sol–gel derived)	electrospun ultrathin nanofibrous scaffold	bone tissue and other	addition of 10 wt % bioglass improved mechanical strength, fiber uniformity, wettability, bioactivity (apatite formation), and cell viability (+47% vs control); demonstrated promising osteogenic potential	*in vitro* (MG-63 osteoblast-like cells)	^133^
PHB	HAp (untreated or silane-treated)	ball milling (mixing), hot pressing	bulk, pressed composites	absorbable fracture fixation material	optimal bending strength at 15 wt % HAp with surface treatment (46.6 MPa); untreated max at 10 wt % HAp (32.74 MPa); silane improved dispersion and interfacial bonding	no biological/cellular model used	^134^
PHB	nano-HAp	salt leaching with compression molding at 180 °C	scaffolds	bone tissue engineering	scaffolds had high porosity (∼77%), interconnected pores (150–300 μm), and compressive strength/modulus in a range of cancellous bone; best mechanical and cell viability at 15 wt % HAp; higher content caused agglomeration and reduced strength	*in vitro* (MG-63 osteoblast-like cells)	^135^
PHB (with chitosan)	nanobioactive glass	electrospinning of polymeric solution with ceramic nanoparticles	electrospun nanofibrous scaffold	dental tissue engineering	PHB/chitosan/bioglass scaffold showed significantly higher cell viability and proliferation than PHB or PHB/chitosan alone; highly biocompatible	*in vitro* (human gingival fibroblasts)	^136^
PHB (with vitamin E)	bioglass	sonication, solvent casting	films with ceramic particles	bone tissue engineering	addition of vitamin E significantly improved wettability, increased protein adsorption (up to +300%), and enhanced MG-63 cell proliferation and attachment; bioglass alone reduced proliferation, but vitamin E counteracted this effect	*in vitro* (MG-63 human osteoblast-like cells)	^137^
PHB, PHB-*co*-6%HV, PHB-*co*-70%4HB, and PHB-*co*-10%3HV-*co*-10%4HB	montmorillonite nanoclays	sonication, solvent casting	films with ceramic nanoparticles	regenerative medicine	P(3HB-*co*-70%4HB)/5% nanoclay showed best improvement in mechanical, thermal, optical, and antimicrobial properties (zone of inhibition against ); performance declined at higher clay content due to agglomeration	*in vitro* antibacterial tests ()	^141^
PHBV	β-TCP/DCPD	heating and dissolving polymer in chloroform, mixing with ceramic and molding into cylinders	pellets (cylindrical)	bone regeneration	compressive strength of 195.2 MPa, Young’s modulus 2050 MPa; bioactive chemical groups detected; *in vivo* confirmed osseointegration and suitability for implantology applications	*in vivo* (New Zealand rabbit bone implantation) tested by the authors in a different study^157^	^143^
PHBV	bioglass	mixing, solvent casting; salt-leaching	macroporous scaffolds	cartilage tissue engineering	bioglass improved hydrophilicity, cell adhesion, migration, extracellular matrix production, and compressive strength; composite formed thicker cartilage-like tissue vs sole polymer	*in vitro* (chondrocytes isolated from New Zealand white rabbits); *in vivo* (subcutaneous implantation in nude mice)	^144^
PHBV	calcium silicate	mixing, solvent casting; salt-leaching	macroporous scaffolds	cartilage tissue engineering	ceramic incorporation enhanced scaffold hydrophilicity, cell adhesion, and migration into the scaffold interior; *in vivo*, composite constructs formed significantly thicker cartilage-like tissue	*in vitro* (chondrocytes isolated from New Zealand white rabbits); *in vivo* (subcutaneous implantation in nude mice)	^145^
PHBV	HAp	injection molding of polymer with ceramic powder, machining	cylindrical specimens	bone replacement	progressive bone formation at the interface; thickness of newly formed bone increased from ∼130 μm (1 month) to ∼770 μm (6 months); direct bone bonding *via* HAp, degradable	*in vivo* (tibial implantation in New Zealand white rabbits	^146^
P(3HB-*co*-3HHx)	HAp nanoparticles	melt-compounding using a co-rotating twin-screw extruder	pellet-sized filaments	bone reconstruction	improved mechanical stiffness; ductility and impact strength decreased; 10 wt % HAp gave the best balance of bone-like properties; good thermal stability up to 260 °C; composites are suitable for low-stress, resorbable bone implants	no biological/cellular model used	^148^
PHB/mcl-PHA blends	β-TCP	foam replica method, sintering, polymer infiltration	highly porous scaffolds	bone, dental, and other tissues applications	composites showed high porosity, increased compressive strength, tailored wettability, and surface roughness; good bioactivity; degradation released hydroxy acids; scaffolds were noncytotoxic, but cell adhesion and proliferation depended on surface hydrophilicity (highest for PHB coating, lowest for 60:40 PHB/mcl-PHA blend)	*in vitro* (MC3T3-E1); SBF immersion test for bioactivity	^149^
PHO	titanium dioxide microfibers	sonication, solvent casting	polymer films with ceramic microfibers	tendon or ligament regeneration	ceramic addition reduced PHO crystallinity but increased stiffness; maintained flexibility; supported cell viability and migration	*in vitro* (MEF 3T3 fibroblasts)	^153^
PHO	TCP	foam replica method, sintering, polymer infiltration	microporous polymer-coated ceramic discs and highly porous scaffolds	bone tissue engineering	composite showed improved mechanical integrity, preserved porosity, promoted apatite layer formation, and released bioactive (*R*)-3-hydroxyacid degradation products	no biological/cellular model used; bioactivity assessment after immersion in SBF	^154^
PHO (physically or chemically modified with diclofenac)	β-TCP	foam replica method, sintering, polymer infiltration	highly porous scaffolds	bone substitutes or wound healing platforms with therapeutic effects	even chemically bonded diclofenac did not show cytotoxicity at low dose; composite supports osteoblast adhesion and penetration	*in vitro* (MC3T3-E1 cells)	^155,156^

The best-known and most extensively studied polyhydroxyalkanoate is PHB, which also takes the lead in composite applications due to its popularity and ease of production. In bone tissue engineering, BioC/PHB composites have already shown promising results. For example, Czechowska et al.^130^ explored PHB combined with silver-decorated βTCP, finding that silver addition enhanced the scaffold’s antibacterial properties, compressive strength, and biocompatibility, supporting its effectiveness in preventing infections and promoting bone regeneration. Skibiński et al.^131^ used porous ceramics doped with silver and silicon, subsequently coated with PHB loaded with gentamicin or ceftazidime. These coatings demonstrated an initial burst followed by sustained antibiotic release from the scaffolds for up to 120 h, as confirmed by noticeable inhibition zones *in vitro*. This dual antibacterial strategy, combining dopants and antibiotic-infused PHB coatings, has the potential to support bone regeneration while effectively combating bacterial infections.

In another study, Iron et al.^132^ examined nanobioactive glass (nBG) incorporated into PHB electrospun scaffolds for bone tissue engineering, reporting that nBG enhanced the mechanical properties and bioactivity of the scaffold, including the formation of an apatite layer on fiber surfaces, indicative of bone bioactivity. Toloue et al.^133^ investigated the addition of magnetic mesoporous bioglass (MMBG) into poly­(3-hydroxybutyrate)-chitosan electrospun nanocomposite scaffolds. They found that MMBG significantly improved mechanical properties, hydrophilicity, and bioactivity, enhancing cell adhesion, proliferation, and biomineralization, thereby making these scaffolds suitable for diverse tissue engineering applications.

Studies by Zhuo et al.^134^ demonstrated that incorporating HAp into PHB improved the tensile strength and Young’s modulus of the composite. Hayati et al.^135^ further developed PHB/nano-HA composites without organic solvents, using powder mixing, compression molding, and NaCl particulate leaching to enhance porosity and pore interconnectivity. Heidary et al.^136^ investigated PHB/chitosan/nBG nanocomposite scaffolds and found improved human gingival fibroblast cell adhesion, proliferation, and viability, suggesting potential applications in dental tissue engineering.

In another approach, Misra et al.^137^ enriched PHB/bioglass composites with vitamin E, which increased sample wettability and hydrophilicity, as shown by protein adsorption studies, and promoted MG-63 cell proliferation. Volkov et al.^138^ developed PHB/HAp composites in desired shapes using a two-stage salt leaching technique with 3D-printed molds. They further filled the scaffolds with an alginate hydrogel containing mesenchymal stem cells (MSCs), demonstrating that these PHB/HAp scaffolds combined with alginate effectively supported MSC growth and osteogenic differentiation, highlighting their potential for bone regeneration applications.

Ding et al.^139^ developed the electrospun PHB/PCL/sol–gel derived silica hybrid scaffolds incorporating levofloxacin that demonstrated biocompatibility, osteoinductivity, and antibacterial properties while also enabling localized drug delivery to prevent postoperative infections. These multifunctional scaffolds could be further adapted for use in craniofacial surgery, cartilage repair, and drug delivery. Also, Nazar et al.^140^ used the potential of polymer–ceramic composites by developing a 3D-printed PHB/dextran/whitlockite scaffold coated with sildenafil-loaded nanofibers, which significantly enhanced osteogenesis and angiogenesis *in vivo*, offering an advanced strategy for craniofacial reconstruction and complex tissue regeneration.

Lastly, Hema et al.^141^ investigated the impact of montmorillonite nanoclays on the physical and biological properties of various PHAs *via* solvent casting. Adding 5 wt % Claytone to P­(3HB-*co*-70%4HB) significantly enhanced mechanical strength, thermal stability, and antimicrobial activity due to strong interfacial bonding and clay dispersion within the polymer matrix. These properties suggest the potential of P­(3HB-*co*-70%4HB)/clay composites for use in regenerative medicine and as environmentally friendly materials.

Another well-studied PHA material is the copolymer PHBV, which has attracted considerable attention as a sustainable alternative to conventional plastics due to its high crystallinity, thermal stability, and ability to degrade naturally. Its mechanical properties can be tailored by adjusting the ratio of 3-hydroxybutyrate (HB) to 3-hydroxyvalerate (HV) units, making PHBV versatile for a range of applications.^142^ PHBV has also been combined with bioceramics for an enhanced biomedical functionality.

Monia^143^ studied a β-TCP/DCPD-PHBV (40/60) composite for bone regeneration, finding that the addition of PHBV improved the mechanical strength and ductility of β-TCP and DCPD, resulting in properties closer to natural bone. The study also showed that the composite’s porosity and rough surface facilitated bone ingrowth and osteoconductivity. In a separate study, Wu et al.^144^ investigated PHBV composites for cartilage tissue engineering by combining PHBV with BG. The incorporation of BG enhanced the mechanical properties, hydrophilicity, and biocompatibility of the scaffolds, which promoted chondrocyte adhesion and proliferation. *In vivo* studies revealed that PHBV/BG composites formed thicker, more homogeneous cartilage-like tissue compared to pure PHBV scaffolds. Wu et al.^145^ also examined PHBV/calcium silicate composite scaffolds for cartilage tissue regeneration, reporting improved cell attachment, infiltration, and growth compared to pure PHBV scaffolds.

Luklinska and Schluckwerder^146^ conducted an *in vivo* study using small cylindrical implants made of HAp (40 vol %) and PHBV implanted into rabbit tibias. The composite promoted new bone formation directly at the implant interface. Over a 6 month period, the implant interface remained active, resulting in a progressive thickening of the new bone layer at the interface. Additionally, collagen-like material was observed being secreted at the interfaces, further supporting the composite’s potential for bone regeneration applications.

Medium-chain-length PHAs, known for their high elasticity, flexibility, and low crystallinity, are well-suited for applications requiring soft and elastomeric materials.^147^ These mcl-PHAs have also been combined with bioceramics to create composite materials for medical applications. For instance, Ivorra-Martinez et al.^148^ studied poly­(3-hydroxybutyrate-*co*-3-hydroxyhexanoate) [P­(3HB-*co*-3HHx)] reinforced with nano-HAp for bone reconstruction. The addition of 20 wt % nano-HAp significantly improved the mechanical strength and thermomechanical resistance of the composite, resulting in properties that more closely match those of native bone than metal alloys or other biopolymers. These findings indicate that P­(3HB-*co*-3HHx)/nano-HAp composites hold promise for low-stress, resorbable, and implantable devices that support bone reconstruction. Skibiński et al.^149^ developed scaffolds based on βTCP and PHB blends with mcl-PHA, finding that the inclusion of mcl-PHA improved the scaffolds’ mechanical properties, hydrophilicity, and bioactivity. Enhanced cell adhesion, proliferation, and viability were observed, underscoring these composites’ potential in dental and bone tissue engineering, as well as in other soft tissue applications.

PHO, a flexible and elastic mcl-PHA, stands in contrast to the more rigid and brittle PHB.^150^ PHO has been primarily applied in soft tissue engineering, such as for arterial prostheses^151^ and heart valves.^152^ When combined with ceramics, PHO demonstrates a further versatility. For example, Malagurski et al.^153^ developed PHO films reinforced with titanium dioxide (TiO_2_) microfibers, resulting in composites with improved stiffness while maintaining flexibility. These PHO/TiO_2_ composites demonstrated high biocompatibility, as confirmed by cell viability and migration studies, suggesting the potential for biomedical applications.

Previous studies have also considered PHO in combination with tricalcium phosphate (TCP) for bone tissue engineering.^154^ Incorporating a macroporous bioceramic core of TCP coated with a thin layer of elastomeric PHO resulted in composites with enhanced mechanical and biological properties. The osteoconductive TCP core supports new bone formation, while the PHO layer not only enhances the stress–strain characteristics but also releases degradation products such as (*R*)-3-hydroxyacids, nourishing the surrounding tissue. Building on this, Skibiński et al.^155^ functionalized porous TCP scaffolds with PHO loaded with diclofenac, resulting in bioactive scaffolds that demonstrated both apatite formation and controlled, sustained drug release. These features suggest that the scaffolds are promising bone substitutes capable of providing localized anti-inflammatory effects. Haraźna et al.^156^ further advanced this concept by modifying β-TCP scaffolds with diclofenac-oligomeric PHO hybrids. This modification significantly improved surface properties, hydrophobicity, and degradation behavior. The resulting composites exhibited excellent biocompatibility with *in vitro* tests confirming their support for cell viability and proliferation, making them viable candidates for therapeutic bone tissue engineering.

### 4.1 Issues Associated with the Use of Biodegradable Polymers in Composites

Despite their many advantages, polymer/bioceramic composites have certain drawbacks that must be considered during the material design and manufacturing. A key issue with implantable composites is their tendency to swell in aqueous environments, leading to excessive volume increase. This can compromise the stability of the implantation area. Additionally, polymers like chitosan, cellulose, and xyloglucan are sensitive to slight changes in pH or temperature, resulting in macroscopic alterations to their structure and, consequently, their properties^158,159^ These fluctuations can occur near the biomaterial shortly after implantation, where inflammation may lower the pH to around 5.4–5.5.^160^ Even more pronounced acidification (as low as pH 3.0) can occur locally during bone remodeling.^161^ Swelling polymers can not only inflame surrounding tissues but also introduce unwanted stresses into the composite. Furthermore, acidification can slow the degradation rate of polymers.^162^

The ideal polymers for these applications are biocompatible and biodegradable, offering the advantage of breaking down and being removed once they have fulfilled their function. The most desirable polymers degrade into nontoxic byproducts that are naturally eliminated from the body *via* metabolic pathways. To initiate degradation, the polymers’ macromolecular structures must be broken down. Various chemical reactions in the body or near the biomaterial can influence the polymer properties and degradation speed. For instance, oxidation, triggered by substances like hydrogen peroxide (H_2_O_2_) and hypochlorite produced by macrophages, neutrophils, and foreign body giant cells, and hydrolysis, induced by ions such as PO_4_^3–^ (from body fluids and bioceramics) or by enzymes, can accelerate degradation. For these reasons, polymer degradation products must not only be nontoxic but also easily removable *via* natural metabolic processes.^163^

Bacteria-derived PHAs are particularly suitable for biomaterials, as their monomers are identical or similar to naturally occurring fatty acids in the body’s fatty acid oxidation pathway. These hydroxy acids are ketone bodies synthesized in liver mitochondria and used by the brain as an energy source.^164^ Moreover, PHA oligomers and monomers stimulate cell proliferation, making them excellent candidates for biomaterial applications.^101^

## 5 Future Perspectives

To date, only a limited number of PHAs have been studied as biomaterials despite the existence of over 100 known PHA monomers. Of these, only eight have found applications in the medical field, primarily due to the limited availability of other monomers.^165^ Production scalability and economic viability also remain significant challenges for these advanced composites. Future research should prioritize improving PHA synthesis efficiency and reducing costs through bioprocess optimization, such as using surplus feedstocks or genetically engineered microbial strains.^166^

The development of BioC/PHA composites holds considerable promise in biomedical engineering, especially for bone tissue engineering and regenerative medicine. However, several challenges and opportunities lie ahead for advancing from laboratory research to clinical application. One primary focus for future research is optimizing the mechanical properties and degradation rates of these composites. While studies show that incorporating bioceramics improves the structural integrity of PHAs, further work is required to fine-tune these properties to more closely mimic natural bone and other targeted tissues, ensuring optimal load-bearing capacity and sustained bioactivity over time. Novel strategies in both the fabrication and clinical deployment of BioC/PHA composites are actively evolving—Panaksri et al.,^167^ for example, introduced a one-pot in situ crystallization method for producing PHB/HAp/TCP microparticles, which not only streamlines processing and improves homogeneity but also achieves a 50% reduction in global warming potential, as confirmed by a comparative life cycle assessment.

Another crucial research area involves enhancing biofunctionalization strategies. Advances in surface modifications—such as incorporating bioactive agents, growth factors, or nanoscale fillers—could significantly improve cell adhesion, proliferation, and differentiation, making scaffolds more effective in supporting tissue regeneration.^168^ Exploring novel PHA blends and copolymers, including mcl-PHAs, may expand the versatility of these materials by offering tailored degradation rates and increased flexibility, which are important for applications in both soft and hard tissues.

Additionally, there is growing interest in developing “smart” BioC/PHA composites that respond to physiological conditions or deliver therapeutic agents in a controlled manner. Integrating drug-loaded polymers, such as diclofenac or antibiotics, into these composites presents the potential for multifunctional implants that not only support bone growth but also provide localized infection or inflammation treatment. This approach aligns with the trend toward personalized medicine, allowing for customization of implants to meet specific therapeutic needs.

Collaboration among academic researchers, industry, and clinical stakeholders will be essential to accelerate the translation of BioC/PHA composites from the laboratory to clinical settings, ensuring that these innovative materials meet regulatory standards and are successfully implemented.

Overall, the future of BioC/PHAs composites appears bright, with the potential to revolutionize regenerative medicine by providing customizable, bioactive, and biodegradable solutions that address current clinical challenges and the increasing demand for sustainable biomaterials.

## 6 Conclusions

In conclusion, combining PHAs, particularly mcl-PHAs, with bioceramics represents a promising approach for creating advanced composite materials that integrate the flexibility, biocompatibility, and biodegradability of PHAs with the structural integrity and bioactivity of bioceramics. This synergy opens new avenues in biomedical applications, such as bone tissue engineering, where the robust mechanical properties of bioceramics complement the adaptable elastomeric qualities of PHAs, enabling a closer mimicry of natural tissue.

These composites can be customized to meet specific clinical needs, improving patient outcomes and expanding the potential of regenerative medicine. Ongoing research into these materials highlights their ability to not only revolutionize medical fields but also contribute to environmental sustainability by reducing dependency on conventional, nondegradable polymers. As the demand for ecofriendly, high-performance materials rises, BioC/PHA composites stand out as a versatile and innovative solution. Embracing these advanced composites could foster substantial progress in both healthcare and sustainable materials science, paving the way for the next generation of biopolymer technologies.

## Data Availability

The authors confirm that the data supporting the findings of this study are available within the article.
